# Neuropeptide regulation of adaptive immunity in the tibia fracture model of complex regional pain syndrome

**DOI:** 10.1186/s12974-018-1145-1

**Published:** 2018-04-11

**Authors:** Wen-Wu Li, Tian-Zhi Guo, Xiaoyou Shi, Frank Birklein, Tanja Schlereth, Wade S. Kingery, J. David Clark

**Affiliations:** 1 Anesthesiology Service, Veterans Affairs Palo Alto Health Care System, Palo Alto, CA USA; 2 Department of Anesthesia, Stanford University School of Medicine, Stanford, CA USA; 3 Palo Alto Veterans Institute for Research, Palo Alto, CA USA; 4 Department of Neurology, University Medical Center, Mainz, Germany; 5 Department of Neurology, DKD Helios Klinik Wiesbaden, Wiesbaden, Germany

**Keywords:** Complex regional pain syndrome, Adaptive immunity, Neuropeptide, Substance P, Calcitonin gene-related peptide, Fracture, Pain

## Abstract

**Background:**

Both dysfunctional neuropeptide signaling and immune system activation are characteristic of complex regional pain syndrome (CRPS). Unknown is whether substance P (SP) or calcitonin gene-related peptide (CGRP) support autoantibody production and, consequently, nociceptive sensitization.

**Methods:**

These experiments involved the use of a well-characterized tibia fracture model of CRPS. Mice deficient in SP expression (Tac1^−/−^) and CGRP signaling (RAMP1^−/−^) were used to probe the neuropeptide dependence of post-fracture sensitization and antibody production. The deposition of IgM in the spinal cord, sciatic nerves, and skin was followed using Western blotting, as was expression of the CRPS-related autoantigen cytokeratin 16 (Krt16). Passive serum transfer to B-cell-deficient muMT mice was used to assess the production of functional autoantibodies in CRPS model mice. The use of immunohistochemistry allowed us to assess neuropeptide-containing fiber distribution and Langerhans cell abundance in mouse and human CRPS patient skin, while Langerhans cell-deficient mice were used to assess the functional contributions of these cells.

**Results:**

Functional SP and CGRP signaling were required both for the full development of nociceptive sensitization after fracture and the deposition of IgM in skin and neural tissues. Furthermore, the passive transfer of serum from wildtype but not neuropeptide-deficient mice to fractured muMT mice caused enhanced allodynia and postural unweighting. Langerhans cells were increased in number in the skin of fracture mice and CRPS patients, and those increases in mice were reduced in neuropeptide signaling-deficient animals. Unexpectedly, Langerhans cell-deficient mice showed normal nociceptive sensitization after fracture. However, the increased expression of Krt16 after tibia fracture was not seen in neuropeptide-deficient mice.

**Conclusions:**

Collectively, these data support the hypothesis that neuropeptide signaling in the fracture limb of mice is required for autoantigenic IgM production and nociceptive sensitization. The mechanism may be related to neuropeptide-supported autoantigen expression.

## Background

Complex regional pain syndrome (CRPS) is a highly enigmatic condition typically affecting a single extremity after surgery or limb trauma. Some resolution of the pain and limb changes is common within a year of onset, but chronic CRPS is a serious problem with over 80% of these patients developing significant disabilities [1]. The occurrence of CRPS after limb surgery involving postoperative splint or cast immobilization is particularly common. The incidence after hand surgery is estimated to be 5–40% [2] and more than 4% after foot and ankle surgeries [3]. Current treatments are of limited efficacy and the basic pathophysiological mechanisms supporting CRPS are still not disentangled, thus constraining the development of effective management. Two potential CRPS mechanisms being extensively investigated are dysfunction of neuropeptide signaling contributing to facilitated neurogenic inflammation, and activation of the innate and adaptive immune systems [4–6]. How these systems might communicate in CRPS, so-called neuroimmune interactions, is unclear at this point.

Evidence from CRPS patients, human volunteers, and laboratory animals suggest that neuropeptide containing sensory primary afferent C-fibers function aberrantly in CRPS resulting in vascular symptoms, trophic changes, and pain [7–9]. Using the mouse and rat fracture-cast immobilization models of CRPS, it has been shown that facilitated release of neuropeptides such as substance P (SP) and calcitonin gene-related peptide (CGRP) from sensory C-fibers [8] leads to inflammation and pain sensitization by the activation of neuropeptide receptors on keratinocytes stimulating expression of high levels of inflammatory cytokines (TNF, IL-1, IL-6) and nerve growth factor (NGF) [8, 10–13], and on mast cells, inducing degranulation [14]. Production of these mediators may underlie the sensitization to noxious stimuli applied to the skin of CRPS patients [15], and the trophic changes sometimes seen in the skin as well [16]. Interestingly, neuropeptides such as SP stimulate Langerhans antigen presenting cell activity in skin either directly or by stimulating the production of cytokines in skin [17].

Growing evidence supports the hypothesis that autoimmunity is involved in CRPS. For example, immunohistochemical studies suggest sympathetic neurons are targets for autoantibodies from some CRPS patients [18]. Other experiments using a cardiomyocyte preparation suggest that a majority of CRPS, but not healthy patients, have autoantibodies binding to and activating M-2 muscarinic, β-2, or α1a adrenergic receptors [19, 20]. Moreover, case reports suggest Langerhans cells are increased in the affected skin of some CRPS patients [21], although patients with very chronic CRPS may have reduced Langerhans cell numbers [22]. Using the tibia fracture-cast model of CRPS, our own laboratory has shown that ablation of B cell populations genetically or by using biologic agents limits CRPS-like changes in fracture animals [23], while the passive transfer of autoantibody-containing serum from fracture animals to B cell-deficient muMT fracture-cast mice recapitulates the nociceptive and inflammatory changes [24]. Efforts to discover the involved autoantigens have identified keratin 16 (Krt16) as a target for autoantibodies from the fracture-cast model as well as CRPS patients [25].

Considering these observations, we set out to determine if neuropeptides produced by primary afferent sensory fibers stimulate the production of autoantibodies which in turn support nociceptive sensitization after tibia fracture and casting. Further, we hypothesized that autoantibody production might rely on Langerhans cell activation, the expression of known autoantigens or both.

## Methods

### Animals

These experiments were approved by the Veterans Affairs Palo Alto Health Care System Institutional Animal Care and Use Committee (Palo Alto, CA) and followed the animal subject guidelines of the International Association for the Study of Pain.

Mice that were homozygous for a disruption of the SP expressing Tac1 (Tac1^−/−^) gene (B6.Cg-Tac1^tm1Bbm^/J), and wildtype (WT) controls (C57Bl/6) were obtained from Jackson Laboratory (004103). CGRP receptor binding requires the receptor activity-modifying protein 1 (RAMP1). Mice that were homozygous for disruption of the RAMP1 gene (RAMP1^−/−^) were generated by Dr. Kazutake Tsujikawa (Dept. Immunology, Osaka University) on a C57Bl/6 background [26]. The Cre-loxP system was used to delete exon 2 of the RAMP1 gene, with splicing of exons 1 to 3 creating a frameshift resulting in a termination codon at the beginning of exon 3. The genotype of each breeding pair was confirmed by extracting DNA from tail snips and performing a PCR assay using an Extract-N-AmpTM kit (XNAT-1KT, Sigma). For serum transfer experiments, muMT mice lacking mature B cells and immunoglobulin, on a C57BL/6J congenic background (Jackson Laboratory, 002288), were employed. Additional experiments utilized Langerhans cell (LC)-deficient mice on a C57BL/6J congenic background (Jackson Laboratory, 017949).

The animals were housed in isolator cages after fracture with solid floors covered with 3 cm of soft bedding, and were given food and water ad libitum. During the experimental period, the animals were fed Lab Diet 5012 (PMI Nutrition Institute, Richmond, IN), which contains 1.0% calcium, 0.5% phosphorus, and 3.3 IU/g vitamin D3, and were kept under standard conditions with a 12-h light-dark cycle.

### CRPS patients

Eight CRPS patients were enrolled at the department of Neurology, University Medical Centre Mainz, Germany. Patients provided informed consent and the investigation was reviewed by the IRB of the Rhineland – Palatinate medical association (837.050.04 (4208)). Inclusion criteria included (1) meeting the new International Association for the Study of Pain clinical diagnostic criteria for CRPS at the time of biopsy [27], (2) unilateral symptoms in the hand or foot, allowing the use of the contralateral limb for control biopsy, and (3) no recent glucocorticoid or bisphosphonate treatments. Table 1 presents the CRPS patient demographic and clinical data. The average patient age was 50 (range 25–82), 62% of the patients were female, the average duration of CRPS was 9.4 weeks (range 2–24), and the average numerical pain score (NPS, average over the last week) was 7.3 (range 3–9).

**Table 1:** Demographic and clinical data from CRPS patients

Subject ID	CRPS duration (weeks)	Age (years)	Gender	Limb	NPS	Allodynia	FX	Etiology
1	12	82	Female	Arm	8	+	+	FX-radius
2	24	33	Male	Arm	8	+	+	FX-radius
3	4	53	Male	Arm	8	+	–	Surgery-metacarpal phalangeal
4	3	67	Female	Arm	8	+	+	FX-phalangeal
5	12	25	Female	Arm	9	–	–	Soft tissue injury-wrist
6	2	44	Male	Arm	7	+	+	FX-middle hand bone V
7	6	58	Female	Arm	7	–	+	FX
8	12	38	Female	Leg	3	+	–	Soft tissue injury-ankle

NPS, numerical pain score, FX, fracture

### Surgery

The tibia fracture with cast immobilization model was generated in 3-month-old male mice. Under isoflurane anesthesia, a hemostat was used to make a closed fracture of the right tibia just distal to the middle of the tibia. Then the hindlimb was wrapped in casting tape (Delta-Lite) so the hip, knee, and ankle were all fixed. The cast extended from the metatarsals of the hind paw up to a spica formed around the abdomen. A window was left open over the dorsal paw and ankle to prevent constriction when post-fracture edema developed [13]. After fracture and casting, the mice were given 2 days of buprenorphine (0.05 mg/kg s.c.) and enrofloxacin (5 mg/kg s.c.) as well as 1.5 ml of normal saline. Some animals received the selective NK1 receptor antagonist LY303870 25 mg/kg/day subcutaneously for 7 days starting at 2 weeks post-fracture. At 3 weeks after surgery, the mice were anesthetized with isoflurane and the cast removed. All mice had union at the fracture site by manual inspection.

### Hind paw allodynia and unweighting

Mechanical allodynia was assayed using nylon von Frey filaments per the “up-down” algorithm as previously described [28]. The mice were placed on wire mesh platforms in clear cylindrical plastic enclosures 10 cm in diameter and 40 cm in height and allowed to acclimate for 15 min. The paw was tested with one of a series of six von Frey hairs ranging from 0.16, 0.4, 0.6, 1, 1.4, to 2 g in a sequentially increasing stiffness manner. The von Frey hair was applied against the hind paw plantar skin at approximately midsole, taking care to avoid the tori pads, and pressed upward to cause a slight bend in the fiber and left in place for 5 s. Withdrawal of or licking the hind paw after fiber application was scored as a response. When no response was obtained, the next stiffest fiber in the series was applied to the same paw; if a response was obtained, a less stiff fiber was applied. Testing proceeded in this manner until four fibers had been applied after the first positive response. Estimation of the mechanical withdrawal threshold by data fitting algorithm permitted the use of parametric statistics for analysis [29]. Preliminary experiments failed to identify differences in von Frey withdrawal thresholds between uninjured WT, Tac1^−/−^, RAMP1^−/−^, and muMT mice.

An incapacitance device (IITC Inc.) was used to measure hind paw unweighting as described previously [13]. The mice were manually held in a vertical position over the apparatus with the hind paws resting on separate metal scale plates to measure weight distribution. The duration of each measurement was 6 s and six consecutive measurements were taken at 10-s intervals. All six readings were averaged to calculate the bilateral hind paw weight-bearing values. Right hind paw weight-bearing data were analyzed as a ratio between the right hind paw weight and the sum of right and left hind paw values ([2*R*/(*R* + *L*)] × 100%) [23].

### Tissue processing and immunofluorescence confocal microscopy

Three weeks post-fracture and intact control mice were euthanized and immediately perfused with phosphate buffered saline (PBS), pH 7.4 followed by 4% paraformaldehyde (PFA) in PBS, pH 7.4, via the ascending aorta; hind paw skin including sub-dermal layers was removed and post-fixed in 4% PFA for 2 h, and then the tissues were treated with 30% sucrose in PBS at 4 °C before embedding in OCT (Sakura Finetek). Following embedding, 20-μm thick slices were made using a cryostat, mounted onto Superfrost microscope slides (Fisher Scientific), and stored at − 80 °C until used for immunohistochemistry as described previously [10]. Briefly, frozen skin sections were permeabilized and blocked with PBS containing 10% donkey serum and 0.3% Triton X-100, followed by exposure to the primary antibodies overnight at 4 °C in PBS containing 2% serum. Upon detection of the first antigen, primary antibody from a different species against the second antigen was applied to the sections and visualized using an alternative fluorophore-conjugated secondary antibody. Sections were then rinsed in PBS and incubated with fluorophore-conjugated secondary antibodies against the immunoglobulin of the species from which the primary antibody was generated. After three washes, the sections were mounted with anti-fade mounting medium (Invitrogen). Images were obtained using confocal microscopy (Zeiss LSM/510 Upright 2 photon; Carl Zeiss) and stored on digital media. With regard to primary antibodies, Rabbit anti-TNF-α (Thermo Fisher Scientific, diluted 1:1000), rabbit polyclonal to CCL2 (Epitomics, diluted 1:100), rabbit polyclonal to GM-CSF (Abcam, diluted 1:200), rat anti-mouse interleukin (IL)-4 (AbD Serotec, diluted 1:25), mouse monoclonal to CGRP (Abcam, diluted 1:1000), rabbit polyclonal to SP (ImmunoStar, diluted 1:1000), rat anti-mouse CD207 (langerin), clone 929F3.01 (Thermo Scientific Pierce, diluted 1:150) were used. Labeling immunofluorescence was performed with anti-rabbit IgG (1:500) conjugated with cyanine dye 3, donkey anti-rat IgG (1:500) conjugated with fluorescein (FITC), or donkey anti-mouse IgG (1:500) conjugated with cyanine dye 3 secondary antibodies (Jackson ImmunoResearch Laboratories), incubated with respective primary antibodies. After three washes, the sections were mounted with anti-fade mounting medium (Invitrogen). For each skin sample, Langerhans cell numbers per 8–10 high power fields were counted in the epidermis. All slides were coded and counted under blinded conditions.

Under local anesthesia, full-thickness human skin 3-mm punch biopsies were obtained from the bilateral hands of eight early CRPS patients. The biopsies were collected from the same location bilaterally in each patient, so the normal (unaffected) side control biopsy was the mirror image of the affected side skin biopsy. Biopsies were fixed in Zamboni’s fixative for 4 h at 4 °C and then rinsed with 0.1 M PB (pH 7.4) and 50% ethanol followed by paraffin embedding, then 6-μm thick slices are cut, mounted onto slides, and placed in a 65 °C oven for 1 h, deparaffinized in xylene, hydrated through graded alcohols to distilled water. Paraffin sections were antigen-retrieved by IHC-Tek Epitope Retrieval Solutions (IHC World) steaming for 40 min, then cooled to room temperature. Sections are permeabilized and blocked with PBS containing 10% donkey serum and 0.3% Triton X-100, followed by exposure to monoclonal mouse anti-CD207 (Abcam, diluted 1:100) overnight at 4 °C in PBS containing 2% serum. The primary antibody was detected using cyanine dye 3 (CY-3)-conjugated donkey anti-mouse IgG (H + C) antibody (Jackson ImmunoResearch Labs, diluted 1:500). Quantitative studies were based on four or more replicates.

Control experiments included incubation of slices in primary and secondary antibody-free solutions both of which led to low-intensity non-specific staining patterns in preliminary experiments (data not shown).

### Popliteal lymph node dissection and size measurement

The popliteal lymph node is embedded in adipose tissue of the popliteal fossa and is spherical. Mice were euthanized by carbon dioxide asphyxiation followed by cervical dislocation, the hair surrounding the foot-draining popliteal lymph nodes was clipped, the skin over the node was incised, and the subcutaneous tissue, fat, and fascia were carefully dissected under microscope. The lymph node sizes (diameters) were measured in millimeters (mm) using a caliper [30], with the average diameter for each lymph node defined as [(short-axis diameter + long-axis diameter)/2].

### Mouse Bio-Plex Luminex cytokine arrays

Mouse hind paw dorsal skin was collected at 3 weeks after fracture and frozen immediately on dry ice. Then skin samples were cut into fine pieces in ice-cold phosphate buffered saline, pH 7.4, containing a cocktail of protease inhibitors (Roche Applied Science) and followed by homogenization using a Bio-Gen PRO200 homogenizer (PRO Scientific). Homogenates were centrifuged at 12,000*g* for 15 min at 4 °C and supernatants were frozen at − 80°C until required for analyzing CCL2, IL-4, TNF-α, and GM-CSF levels. An aliquot was subjected to protein assay (Bio-Rad) to normalize mediator levels. Mouse Bio-Plex Luminex cytokine array was performed in the Human Immune Monitoring Center at Stanford University. Assay kits were purchased from Affymetrix and used according to the manufacturer’s recommendations. Briefly, samples were mixed with antibody-linked polystyrene beads on 96-well filter-bottom plates and incubated at room temperature for 2 h followed by overnight incubation at 4 °C. Room temperature incubation steps were performed on an orbital shaker at 500–600 rpm. Plates were vacuum filtered and washed twice with wash buffer, then incubated with biotinylated detection antibody for 2 h at room temperature. Samples were then filtered and washed twice as above and suspended in streptavidin-PE. After incubation for 40 min at room temperature, two additional vacuum washes were performed, and the samples re-suspended in Reading Buffer. Plates were read using a Luminex-200 instrument with a lower bound of 100 beads per sample per cytokine. All samples were run in duplicate. Standard curves for each of the analyzed cytokines were included in the run and sample concentrations were automatically calculated.

### Western blot analysis

Western analysis for IgM deposition in skin and neural tissues was performed as described previously [24]. Mouse hind paw skin, sciatic nerve, and spinal cord were harvested at 3 weeks after fracture and stored at − 80 °C. All tissues were homogenized in ice-cold Tris buffer with 0.7% (*v*/*v*) β-mercaptoethanol and 10% glycerol. Lysates were centrifuged at 13,000*g* for 15 min at 4 °C, and the protein concentration of the supernatant was measured by a Bio-Rad DC protein assay reagent (Bio-Rad). Equal amounts of protein (50 μg) were size fractionated by SDS–PAGE and transferred onto a polyvinylidene difluorided membrane. The blots were blocked overnight with 5% normal serum in Tris-buffered saline with 0.5% Tween-20 (TBST) and incubated with primary antibodies against IgM or β-actin (Santa Cruz Biotechnology) for 1 h on a rocking platform at room temperature. After washing in TBST, the blots were incubated with secondary antibody for 1 h at room temperature. The membrane was then washed again, and proteins were detected using ECL chemiluminescence reagent (GE Healthcare). The band intensity was analyzed using National Institutes of Health ImageJ.

Western analysis for cytokeratin 16 (Krt16) expression in hindlimb skin was performed similarly. Blots were blocked overnight with 5% non-fat dry milk in Tris-buffered saline with 0.5% Tween-20 (TBST), then incubated with primary antibodies against Krt16 or β-actin (Santa Cruz Biotechnology) overnight at − 4 °C. After washing in TBST, the blots were incubated with HRP-conjugated secondary antibody (Santa Cruz Biotechnology) for 1 h at room temperature. The membranes were then washed again, and proteins were detected using ECL plus chemiluminescence reagent (Thermo Scientific). Images were obtained using ChemiDoc MP Imaging Systems (Bio-Rad) and analyzed using National Institutes of Health ImageJ.

### Serum transfer experiments

The transfer of serum from WT, Tac1^−/−^, and RAMP1^−/−^ mice into fractured muMT mice was performed as described recently [24]. Briefly, whole blood was collected by transcardial puncture in isoflurane anesthetized 3-week post-fracture mice. After 60 min at room temperature to allow clotting, the samples were centrifuged at 1500*g* for 15 min at 4 °C and the serum supernatants were aliquoted and frozen at − 80 °C until use. The muMT mice had baseline behavioral testing and then underwent right tibia fracture and casting. At 3 weeks post-fracture, the casts were removed and behavioral testing was repeated the next day. Following the behavioral measurements, the muMT mice were injected with the 3-week post-fracture serum previously collected from either WT, Tac1^−/−^, or RAMP1^−/−^ mice (500 μl, i.p.). Behavioral testing was then performed at 1, 7, 14, and 21 days after injection.

### Clodronate treatments

Langerhans cells and other phagocytic cells (macrophages and dendritic cells) were depleted using liposomal clodronate (FormuMax Scientific Inc., Sunnyvale, CA). Mice were either injected intravenously into a tail vein under isoflurane anesthesia with 50 mg/200 μl liposomal clodronate or control liposomes, repeated four times during the fracture period, or were injected intradermally with 50 mg/200 μl liposomal clodronate divided between 3 and 4 different sites in the fracture limb, repeated four times during the fracture period. The first injection was the day before fracture, and the animals were injected weekly thereafter.

### Statistical analysis

All experiments were performed in blinded fashion when feasible. Statistical analysis was performed using a one-way or two-way analysis of variance (ANOVA) with Bonferroni correction for post hoc comparisons. For simple comparisons of two means, an unpaired Student *t* test was performed. All data are presented as the mean ± standard error of the mean (SEM), and differences are considered significant at a *P* value less than 0.05 (Prism 5, GraphPad Software, San Diego, CA).

## Results

### The contribution of neuropeptide signaling to post-fracture hindlimb nociceptive changes

**Fig. 1:**
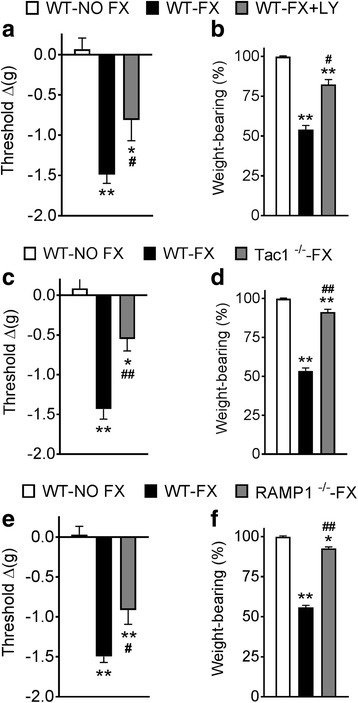
Neuropeptide signaling contributes to the development of evoked and spontaneous post fracture pain behaviors. Wildtype fracture (WT-FX) mice developed hind paw von Frey allodynia and hindlimb unweighting at 3 weeks post-fracture, compared to control wildtype no fracture (WT-NO FX) mice, but wildtype FX mice treated with the substance P (SP) tachykinin 1 receptor antagonist LY303870 (WT-FX + LY) had reduced allodynia (**a**) and unweighting (**b**). Substance P-deficient fracture mice (Tac1^−/−^-FX, panels **c, d**), and calcitonin gene-related peptide (CGRP) receptor-deficient fracture mice (RAMP1^−/−^-FX, panels **e, f**) also exhibited attenuated allodynia and unweighting after fracture. These results support the hypothesis that neuropeptide signaling contributes to the development and maintenance of pain behaviors after fracture. Measurements for **a**, **c**, and **e** represent the difference between the fracture side and contralateral paws, thus a negative value represents a decrease in mechanical nociceptive thresholds on the affected side. Measurements for **b**, **d**, **f** represent weight-bearing on the fracture hindlimb as a ratio to 50% of bilateral hindlimb loading, thus, a percentage lower than 100% represents hind paw unweighting. Data were analyzed using a one-way analysis of variance (ANOVA) with Bonferroni correction test for post hoc contrasts, error bars indicate SEM, *n* = 8 per cohort. *P < 0.05, **P < 0.01 vs. control WT-NO FX, #*P* < 0.05, ##*P* < 0.01 vs WT-FX

Previously, we have established that in the intact unfractured mice there were no differences between wildtype (WT), SP-deficient (Tac1^−/−^), and CGRP receptor-deficient (RAMP1^−/−^) mice in terms of hind paw von Frey thresholds [13]. In this study, as shown in Fig. 1, wildtype fracture (WT-FX) mice developed hind paw allodynia and unweighting at 3 weeks post-fracture, but wildtype FX mice treated with the substance P (SP) tachykinin 1 receptor antagonist LY303870 (WT-FX + LY) had reduced allodynia and unweighting (Fig. 1a, b). Substance P-deficient (Tac1^−/−^) mice (Fig. 1c, d) and calcitonin gene-related peptide (CGRP) receptor signaling-deficient (RAMP1^−/−^) mice (Fig. 1e, f) also exhibited attenuated allodynia and unweighting after fracture. These results support the hypothesis that neuropeptide signaling contributes to the development and maintenance of pain behaviors after fracture.

### Neuropeptide signaling contributes to IgM deposition in the skin, sciatic nerve and spinal cord after fracture

**Fig. 2:**
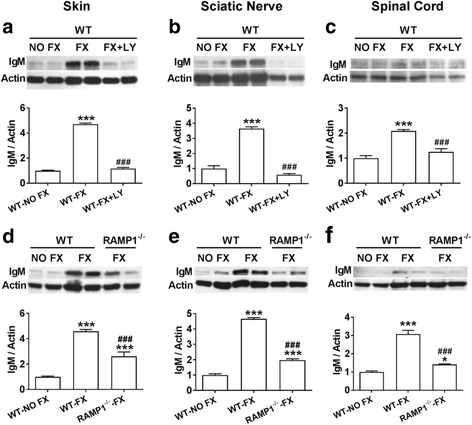
Neuropeptide signaling is required for post fracture immunoglobulin M (IgM) deposition in the skin, sciatic nerve, and spinal cord. IgM protein levels in mouse hind paw skin, sciatic nerve, and spinal cord at 3 weeks after fracture were determined by Western blot analysis. Compared to control wildtype no fracture mice (WT-NO FX), IgM levels in the skin, sciatic nerve, and spinal cord from wildtype fracture mice (WT-FX) were dramatically increased at 3 weeks post fracture (**a–f**). Treating fracture mice with the substance P tachykinin1 receptor antagonist LY303870 (FX + LY) for 7 days just prior to cast removal reversed IgM-antigen complex deposition in all tissues (**a–c**). IgM levels in skin, sciatic nerve, and spinal cord were also elevated in the CGRP receptor-deficient fracture mice (RAMP1^−/−^-FX) at 3 weeks post fracture compared with WT-NO FX control mice, but this increase was attenuated compared to the increase observed in the WT-FX mice (**d–f**). Data were analyzed using a one-way analysis of variance (ANOVA) with Bonferroni correction test for post hoc contrasts, data are expressed means ± SEM, *n* = 6 per cohort. ***P < 0.001 vs. control WT-NO FX, ^###^*P* < 0.001 for FX + LY or RAMP1^−/−^-FX vs WT-FX

Previous reports of SP and CGRP supporting nociceptive sensitization in the mouse fracture model [13, 31] and the essential role of B cell activation and autoantibody formation in post-fracture sensitization [23–25] prompted us to ask whether neuropeptide signaling was required for IgM autoantibody formation. Western immunoblots were used to evaluate the IgM levels in mouse skin, sciatic nerve, and spinal cord from WT no fracture controls (WT-No FX), WT fractured mice (WT-FX), WT fractured mice with pharmacological blockade of SP signaling (WT-FX + LY), and CGRP receptor-deficient FX mice (RAMP1^−/−^-FX). Compared to no fracture control mice, the 3-week post-fracture WT mice exhibited two- to fourfold IgM increases in all tissues (Fig. 2a–f). Previously, we demonstrated that this increase in IgM antibody-antigen deposition in skin, nerve, and cord is restricted to the fracture limb and corresponding lumbar cord with peak deposition occurring between 12 and 18 weeks after fracture and resolving by 23 weeks after fracture, corresponding to the time course for the development and resolution of nociceptive sensitization after tibia fracture and casting in mice [24]. To explore the ways neuropeptides might be involved in fracture-induced IgM deposition, we treated mice with the tachykinin 1 receptor antagonist LY303870 for 7 days starting at 2 weeks post-fracture. LY303870 treatment completely inhibited fracture-induced IgM increases in mouse skin, sciatic nerve, and spinal cord (Fig. 2a–c). Furthermore, fracture RAMP1^−/−^ mice lacking functional CGRP receptors had attenuated levels of IgM in the three tissues analyzed (Fig. 2d, e). These results suggest that neuropeptide signaling contributes to post-fracture changes of IgM in mouse skin, sciatic nerve and spinal cord.

### Passive transfer experiments

**Fig. 3:**
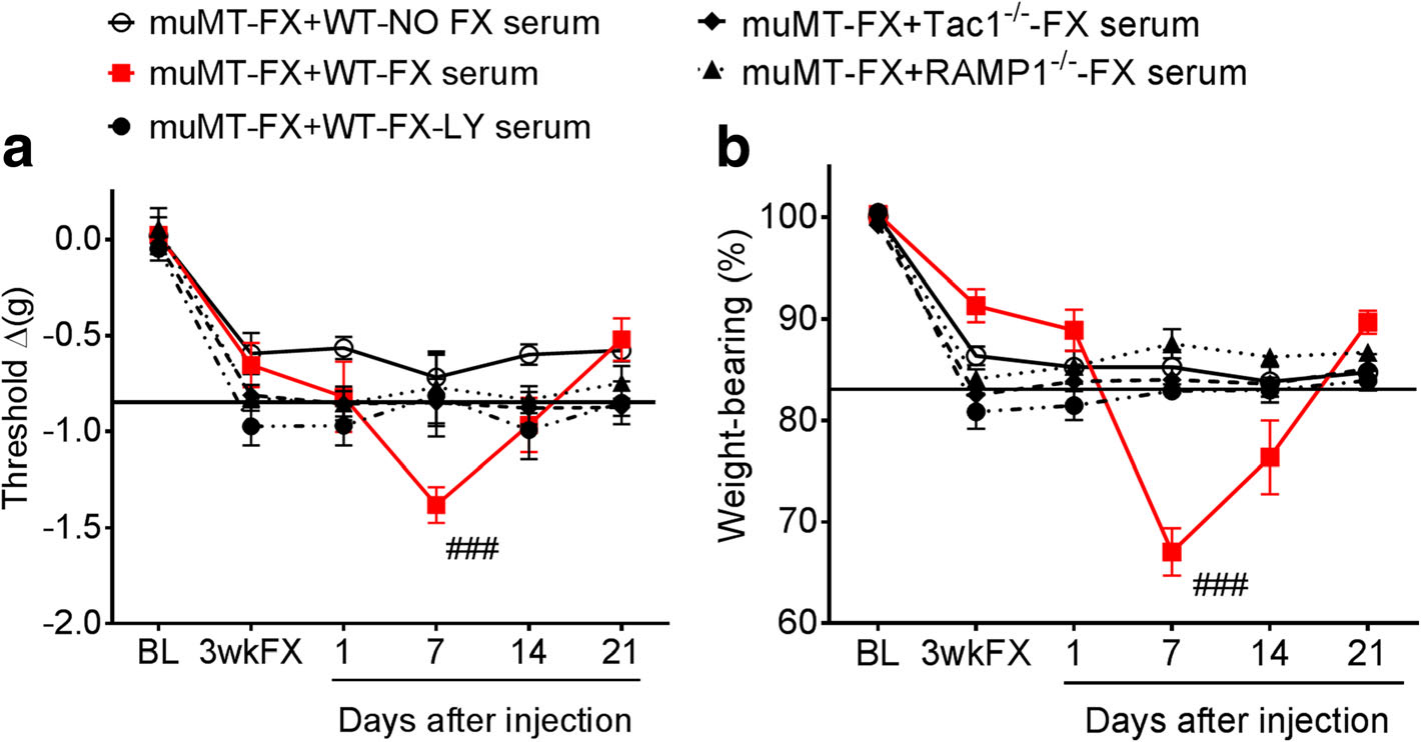
Neuropeptide signaling is crucial for the development of post fracture pronociceptive serum effects. When serum from 3 week post fracture wildtype fracture mice (WT-FX) was injected into 3 week post fracture muMT fracture mice (muMT-FX), the mice gradually developed increased hind paw von Frey allodynia (**a**) and unweighting (**b**) over the ensuing week that is resolved by 2 weeks post-injection, as compared with muMT mice receiving wildtype no fracture (WT-NO FX) mouse serum. When serum from fracture mice treated with the substance P (SP) tachykinin 1 receptor antagonist LY303870 for 7 days (WT-FX-LY) was injected in to the muMT-FX mice, there was no pronociceptive effect. Similarly, when serum from 3-week post fracture mice lacking SP (Tac1^−/−^-FX) or the calcitonin gene-related peptide receptor (RAMP1^−/−^-FX) was injected into 3-week post fracture muMT-FX mice, there was no effect on hind paw allodynia (**a**) and unweighting (**b**). Data were analyzed using a two-way repeated measures analysis of variance (ANOVA) with Bonferroni correction test for post hoc contrasts, data are expressed means ± SEM, *n* = 8–10 per cohort. ^###^*P* < 0.001 for muMTFX-FX + WT-FX serum vs muMT-FX + WT-NO FX serum

Based on the behavioral and biochemical data, a set of experiments was designed to test the hypothesis that intact neuropeptide signaling is required to produce pro-nociceptive autoantibodies after fracture and casting in mice. As shown in Fig. 3, when serum from 3-week post-fracture WT fracture mice was injected into 3-week post-fracture B cell-deficient muMT fracture mice (muMT-FX + WT-FX), the muMT mice gradually developed increased hind paw von Frey allodynia and unweighting over the ensuing week. And, consistent with the serum half-life of IgM, these pronociceptive effects resolved by 2 weeks post-injection. Serum from non-fracture WT mice (muMT-FX + WT-No FX) had no effects (Fig. 3). When 3-week post-fracture muMT fracture mice were injected with serum from 3-week post-fracture Tac1^−/−^ mice (muMT-FX+ Tac1^−/−^-FX) or 3-week post-fracture RAMP1^−/−^ mice (muMT-FX+ RAMP1^−/−^-FX), there was no effect on hind paw allodynia or unweighting. In addition, the passive transfer of serum from LY303870-treated WT fracture mice to the fracture muMT mice (muMT-FX + WT-FX-LY) had no significant effect on nociception. These data suggest that fracture-induced autoantibodies require intact neuropeptide signaling.

### Intimate association of SP- and CGRP-containing epidermal nerves with Langerhans cells

**Fig. 4:**
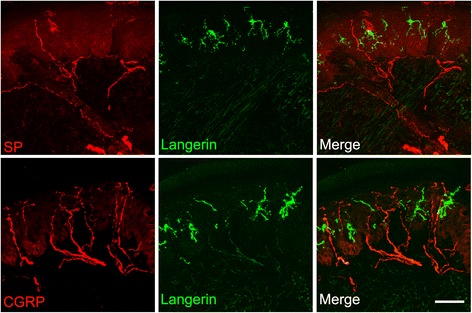
Langerhans cells associate intimately with epidermal substance P (SP) and calcitonin gene-related peptide (CGRP) immunostained sensory axons in the hind paw skin collected 3 weeks after tibia fracture. Top panels are double immunohistochemical staining of SP (red) and CD-207 (Langerin, a Langerhans cell marker, green) in a dorsal hind paw skin section, bottom panels are immunostaining of CGRP (red) and langerin (green) in a dorsal hind paw skin section. These representative photomicrographs demonstrate the close approximation of the SP and CGRP containing sensory afferent neurons and the Langerhans cells in the epidermis. Scale bar = 20 μmLangerhans cells associate intimately with epidermal substance P (SP) and calcitonin gene-related peptide (CGRP) immunostained sensory axons in the hind paw skin collected 3 weeks after tibia fracture. a Double immunohistochemical staining of SP (red) and CD-207 (Langerin, a Langerhans cell marker, green) in a dorsal hind paw skin section. b Immunostaining of CGRP (red) and langerin (green) in a dorsal hind paw skin section. These representative photomicrographs demonstrate the close approximation of the SP and CGRP containing sensory afferent neurons and the Langerhans cells in the epidermis. Scale bar = 20 μm

Langerhans cells (LCs) are dendritic cells that reside in the basal and suprabasal layers of the epidermis. When activated, LCs extend their dendrites as a part of the process by which they acquire foreign antigens. These cells specialize in antigen presentation and represent an important component of skin serving as epidermal sentinels of the adaptive immune system [32]. Intradermal nerve fibers represent terminations of dorsal root ganglion sensory neurons serving a variety of functions including nociception and neurogenic inflammation [33]. Clinical observations suggest that the nervous and immune systems are closely related; neural reflexes regulate both innate and adaptive immunity [34, 35]. The above findings prompted us to examine the histological association between neuropeptide-containing sensory nerves and LCs by immunohistochemistry. Figure 4 presents representative confocal microscopy of immunofluorescence staining for SP and CGRP containing sensory nerve fibers along with the LC marker CD207 (Langerin) in dorsal hind paw skin at 3 weeks after fracture. These images demonstrate that the cutaneous peptidergic nerve fibers are intimately associated with LCs in the epidermis.

### Neuropeptide signaling mediates the post-fracture increases in Langerhans cell (LC) activity in the skin

**Fig. 5:**
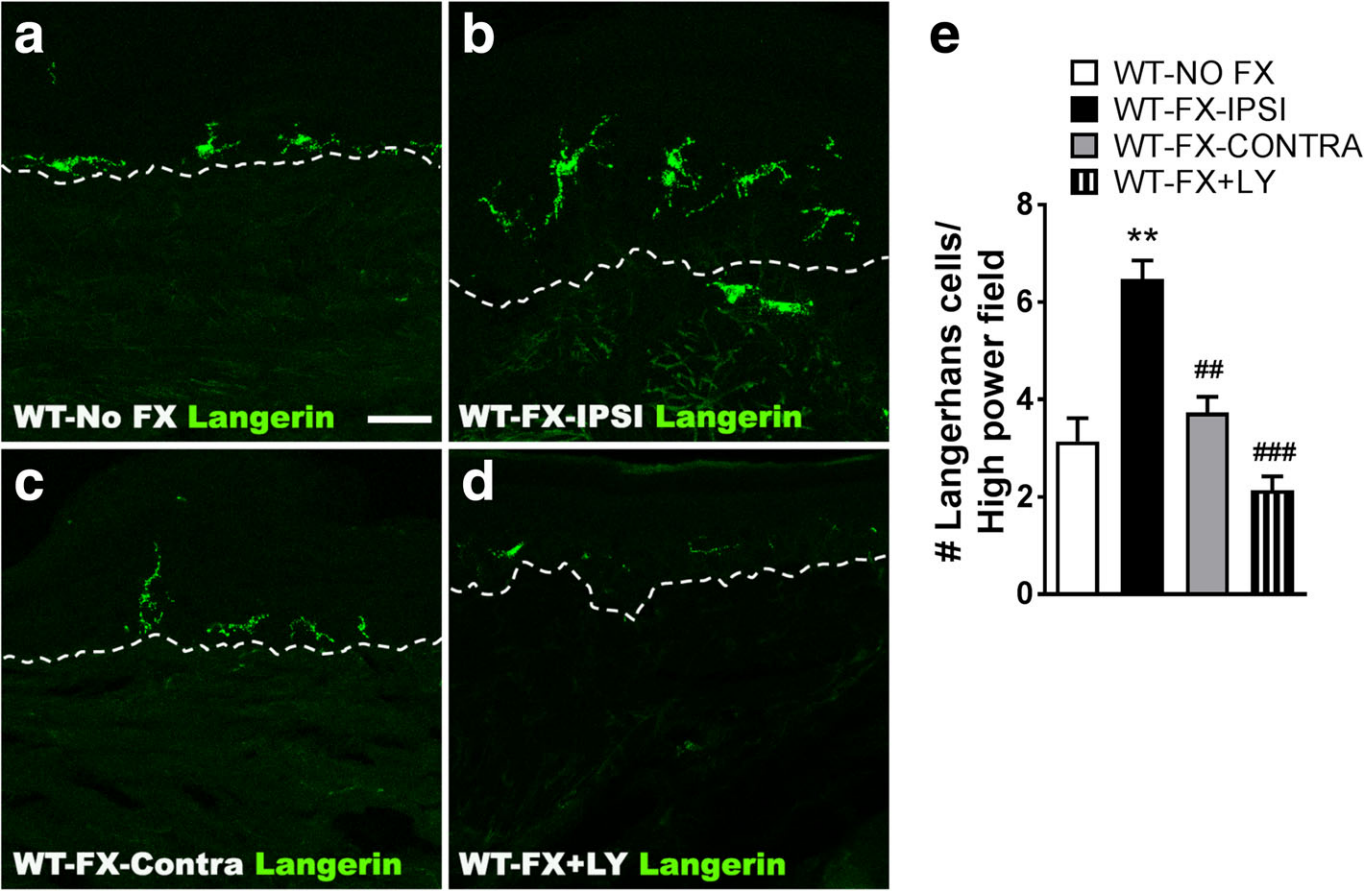
Substance P signaling perpetuates the post fracture increase in Langerhans cell (LC) numbers observe in the injured hindlimb skin. Fluorescent immunohistochemical staining of langerin, a LC marker (green), in hind paw skin sections from the control wildtype no fracture mice (**a**, WT-NO FX) and from the ipsilateral (**b**, WT-FX-IPSI) and contralateral (**c**, WT-FX-CONTRA) hind paws of fracture mice at 3 weeks post-injury. After fracture, the epidermal LC numbers increased and cell morphology was altered, with the cells growing larger with increased dendritic branching, compared to the control or the contralateral side. Some LCs appeared to be migrating through the epidermal basement membrane (dashed lines). Daily treatment for 7 days (30 mg/kg/day) with the SP tachykinin1 receptor antagonist LY303870 (**d**, WT-FX + LY) completely reversed the fracture induced increase in LC numbers. Scale bar = 30 μm. **e** Quantification of hind paw skin Langerhans cells in tissue sections from no fracture controls, fracture ipsilateral and contralateral hind paws, and fracture mice treated with LY303870. There was a twofold ipsilateral increase in hind paw skin LC numbers after fracture, as compared to the controls, and LY303870 treatment blocked this increase. Data were analyzed using a one-way analysis of variance (ANOVA) with Bonferroni correction test for post hoc contrasts, error bars indicate SEM, *n* = 6 per cohort. **P < 0.01 for vs. WT-NO FX, ##*P* < 0.01, ###*P* < 0.001 vs. WT-FX-IPSI

We sought to determine if there was post-fracture activation of LCs and whether neuropeptide signaling modulates this activation. Figure 5a–d shows representative immunostaining for Langerin in the epidermis of plantar hind paw skin from WT-No FX control mice, from ipsilateral and contralateral hind paw of WT-FX mice at 3 weeks post-injury, and from ipsilateral hind paw of wildtype fracture mice treated with LY303870 (WT-FX + LY). After fracture, the epidermal LC morphology was altered, with the cells becoming larger and showing increased dendritic branching. LC numbers also increased, compared to the control or the contralateral side. Some LCs appeared to be migrating through the epidermal basement membrane. Daily treatment for 7 days with the tachykinin 1 receptor antagonist LY303870 completely abolished the fracture-induced increase in LC numbers (Fig. 5e). These results indicate that SP signaling contributes to LC activation after fracture.

**Fig. 6:**
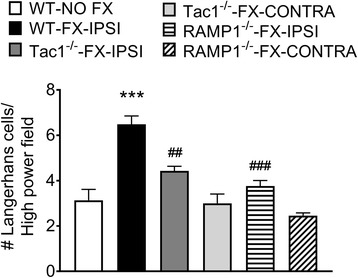
Substance P (SP) and calcitonin gene-related peptide CGRP) signaling is required for the post fracture increase in Langerhans cell (LC) numbers. Glabrous hind paw skin was harvested from 3-week post fracture wildtype mice (WT-FX-IPSI), fracture mice lacking SP (Tac1^−/−^-FX-IPSI and Tac1^−/−^-FX-CONTRA), and fracture mice lacking the calcitonin gene-related peptide receptor (RAMP1^−/−^-FX-IPSI and RAMP1^−/−^-FX-CONTRA), with wildtype no fracture mice (WT-NO FX) serving as controls. Immunohistochemical quantification demonstrated a 206% increase in epidermal LC numbers in the WT-FX-IPSI vs WT-NO FX controls, whereas Tac1^−/−^-FX-IPSI mice had 68% less LCs than the WT-FX-IPSI mice. Similarly, RAMP1^−/−^-FX-IPSI mice had 78% less LCs than the WT-FX-IPSI mice. Data were analyzed using a one-way analysis of variance (ANOVA) with Bonferroni correction test for post hoc contrasts, error bars indicate SEM, *n* = 5-6 per cohort. ***P < 0.001 for vs. Control, ## *P* < 0.01, and ###*P* < 0.001 vs. FX-IPSI-WT.

The mechanistic role of sensory neuropeptides after fracture in modulating LC response was further evaluated using Tac1^−/−^ and RAMP1^−/−^ mice. Wildtype, Tac1^−/−^, and RAMP1^−/−^ mice underwent tibia fracture and casting for 3 weeks, then the cast was removed and hind paw skin was collected for LC analysis. As shown in Fig. 6, the numbers of epidermal LCs increased by 206% in hind paw skin ipsilateral to the fracture in WT-FX mice, compared to the WT-No FX controls, whereas *Tac1*^−/−^-FX mice had 68% less LCs than the WT-FX. *RAMP1*^−/−^-FX mice had 58% less LCs than the WT-FX mice.

### Epidermal Langerhans cell number increased in the patients with CRPS

**Fig. 7:**
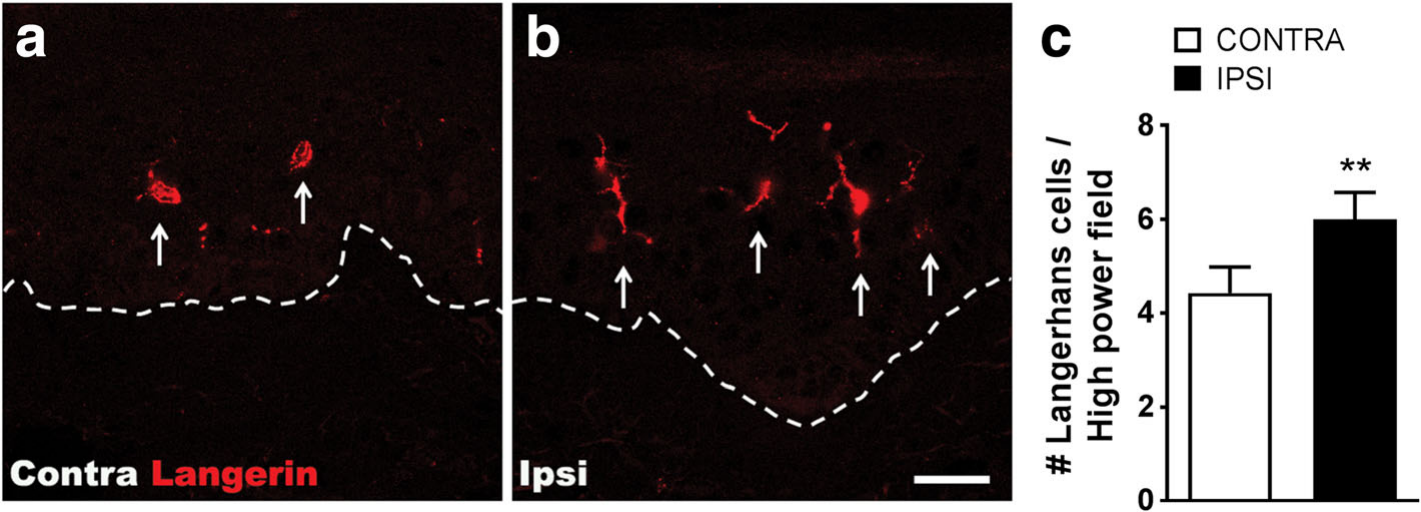
Langerhans cell (LC) numbers were increased in the affected skin of early CRPS patients. Fluorescent immunohistochemical staining of Langerin, an LC marker (red) in dorsal hand skin biopsies collected from the contralateral (**a**) and CRPS affected (**b**) sides. **c** Similar to the changes observed in the fracture mice, the number of epidermal LCs increased 40% in the CRPS-affected skin (IPSI), compared to the contralateral (CONTRA) side. Langerhans cells in CRPS-affected skin also exhibited exaggerated ramified processes suggesting cellular activation and differentiation. Dashed lines represent the epidermal-dermal border. Data were analyzed using paired Student’s *t* test, error bars indicate SEM, *n* = 8 per cohort. **P < 0.01 for vs. contralateral. Scale bar = 20 μm

Next, we evaluated whether CRPS patients also exhibited increased epidermal LC number in the affected skin. Using bilateral CRPS skin biopsy samples, we observed an increase in epidermal LC numbers in the affected skin of early (average duration 9.4 weeks) CRPS patients, compared to the contralateral side. Some Langerhans cells in CRPS-affected skin also exhibited exaggerated ramified processes suggesting cellular activation and differentiation (Fig. 7a, b).

### Increased levels of CCL2, IL-4, GM-CSF, and TNF in the skin after fracture

**Fig. 8:**
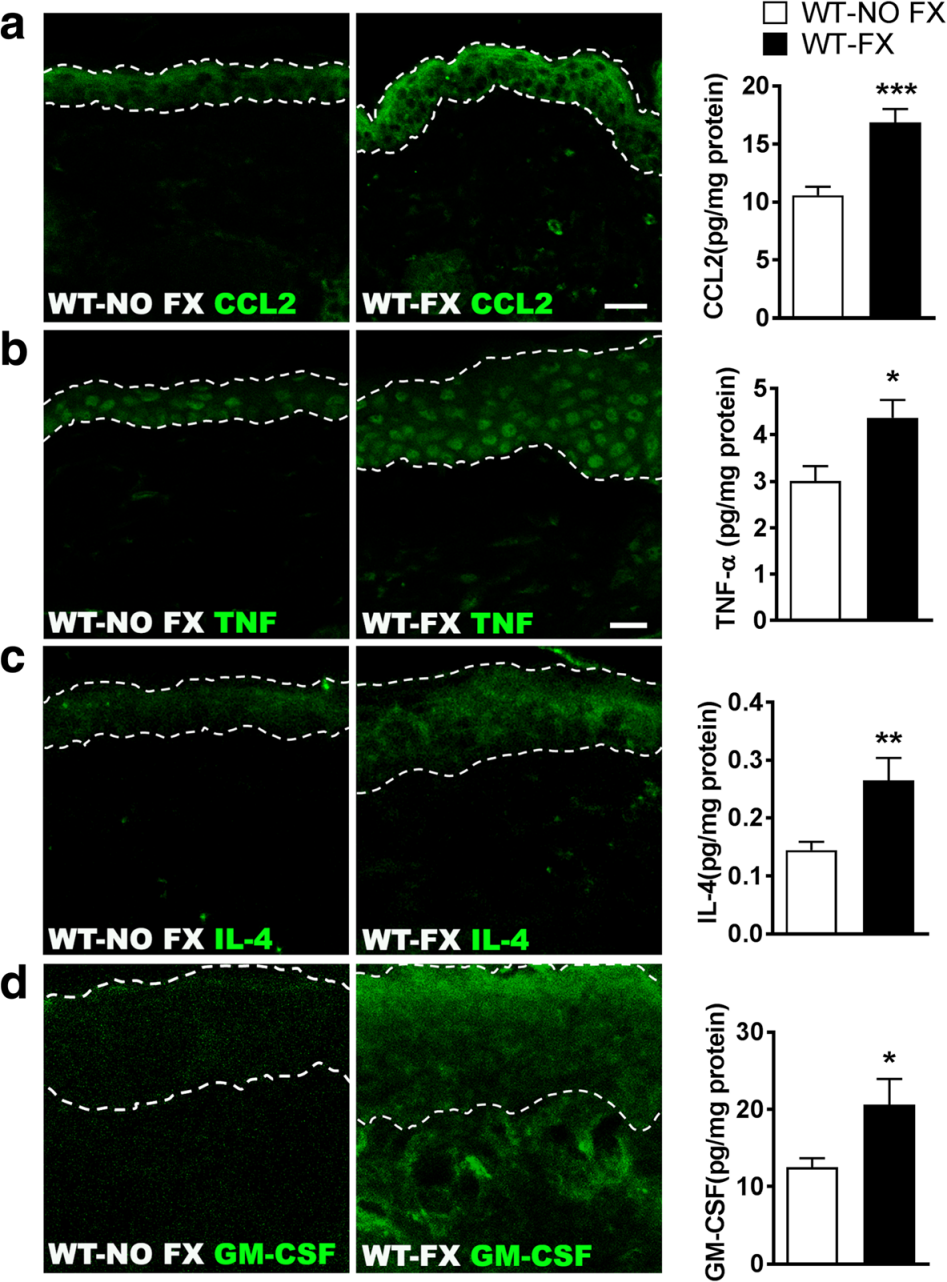
Hind paw epidermal inflammatory chemokine and cytokine levels were increased in wildtype mice fracture (WT-FX) vs no fracture controls (WT-NO FX). At 3 weeks post fracture, elevated levels of **a** chemokine (C–C motif) ligand 2 (CCL2), **b** tumor necrosis factor-α (TNF), **c** interleukin-4 (IL-4), and **d** granulocyte-macrophage colony-stimulating factor (GM-CSF) proteins were observed in the hind paw epidermis by immunohistochemistry and confirmed by Bio-Plex Luminex cytokine arrays. The dotted lines define the limits of the epidermis. Data were analyzed using unpaired Student’s *t* test, error bars indicate SEM, *n* = 8 per cohort. ^*^P < 0.05, **P < 0.01, and ***P < 0.001 vs WT-NO FX. Scale bar = 20 μm

Under normal conditions, LCs can replicate locally in the epidermis, but in inflammatory states, LCs are recruited from circulating monocyte progenitor cells to the epidermis by local expression of the chemokine CCL2 (monocyte chemoattractant protein 1, MCP-1), whereupon they mature into LCs if exposed to other stimulatory cytokines [36]. Therefore, immunohistochemistry and cytokine arrays were used to assess the protein levels of CCL2, IL-4, GM-CSF, and TNF in mouse skin from intact control, and 3-week post-fracture mice. As shown in Fig. 8, at 3 weeks post-fracture, there was upregulation of keratinocyte CCL2 expression and elevated post-fracture levels of the cutaneous cytokines required for the recruitment, proliferation, and differentiation of fully functional antigen presenting LCs.

### Popliteal lymph node enlargement was observed after fracture

LCs are the prototypic antigen-presenting dendritic cells residing in the epidermis, which acquire antigens in the peripheral tissue and transport them to regional lymph node following stimulation by cutaneous injury, infection, or in other disease states [37, 38]. Popliteal and iliac lymph node enlargement has been previously observed at 7 days after tibia fracture and casting in rats [39] and after tibia fracture in man [40]. Lymph nodes from a number of mammalian species have been reported to possess SP and CGRP nerve fibers that travel as individual fibers in the parenchyma of the cortex and medulla among lymphocytes.

**Fig. 9:**
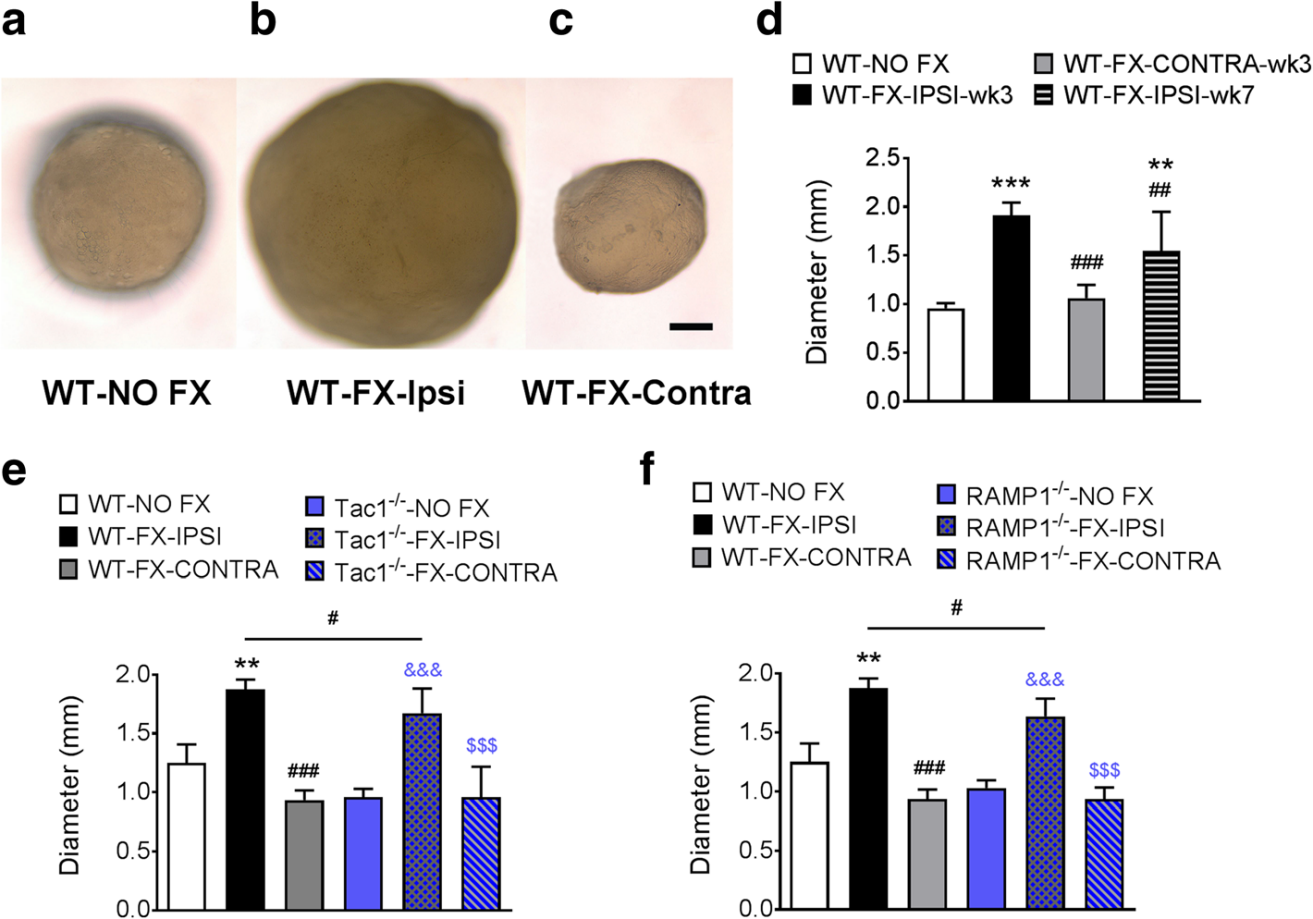
Neuropeptide signaling minimally contributed to post fracture popliteal lymphadenopathy. Changes in size of the popliteal lymph node at 3 weeks after fracture (FX) in wildtype (WT), SP-deficient (Tac1^−/−^), and CGRP receptor-deficient (RAMP1^−/^) mice. Representative popliteal lymph node images are presented from an **a** intact wildtype (WT-NO FX), **b** ipsilateral (WT-FX-IPSI), and **c** contralateral (WT-FX-CONTRA) to fracture in a wildtype mouse. **d** At 3 weeks post fracture, there was 97% increase in lymph node diameter (WT-FX-IPSI-wk3) vs WT-NO FX controls (1.9 ± 0.1 mm vs 0.96 ± 0.1 mm) and this increase persisted at 7 weeks after fracture (WT-FX-IPSI-wk7, 1.5 ± 0.4 mm). **e** The increase in popliteal lymph node size in substance P-deficient fracture mice (Tac1^−/−^-FX-IPSI) 11% less than that of WT-FX-IPSI. **f** The increase in popliteal lymph node size in CGRP receptor-deficient fracture mice (RAMP1^−/^-FX-IPSI) was 16% less than that of WT-FX-IPSI. Data were analyzed using a one-way analysis of variance (ANOVA) with Bonferroni correction test for post hoc contrasts, error bars indicate SEM, *n* = 6 per cohort. **P < 0.01, ***P < 0.001 vs. WT-NO FX, # *P* < 0.05, ##*P* < 0.01 and ###*P* < 0.001 vs. FX-IPSI-wk3 or WT-FX-IPSI, &&& P < 0.001 vs. the respective unfractured control mice (i.e., either Tac1^−/−^-NO FX or RAMP1^−/−^-NO FX), $$$ *P* < 0.001 vs. the respective fractured knockout mice (i.e., either Tac1^−/−^- FX-IPSI or RAMP1^−/−^- FX-IPSI). Scale bar = 250 μm

We therefore examined the sizes of popliteal lymph nodes after fracture and the contribution of neuropeptide signaling. Wild type, Tac1^−/−^, and RAMP1^−/−^ *mice* underwent a right distal tibia *fracture* and cast immobilization for 3 weeks, then the animals were sacrificed and the popliteal lymph nodes were harvested under a dissecting microscope. The lymph nodes were then measured using a caliper. Another group of lymph nodes were harvested from a second cohort of mice at 7 weeks after fracture. As shown in Fig. 9a–c, tibia fracture was accompanied by significant enlargement of popliteal lymph nodes 3 weeks after fracture. Figure 9d reveals the average diameter of popliteal lymph nodes was 198%, and 156% greater at 3 and 7 weeks after fracture respectively (1.90 ± 0.13 mm in 3-week post-fracture *n* = 6 vs 0.96 ± 0.05 mm in control groups, *n* = 6; 1.50 ± 0.40 in 7-week post-fracture *n* = 9 vs 0.96 ± 0.05 mm in control groups, *n* = 6). Furthermore, we observed that the enlargement of popliteal lymph nodes in neuropeptide-deficient fracture mice was slightly but significantly less than that of wildtype fracture mice (Fig. 9e, f). These findings suggest that the lymph node reaction to tibia fracture is partially responsive to neuropeptide signaling.

### Langerhans cells (LCs) do not mediate the development of nociceptive changes after fracture

**Fig. 10:**
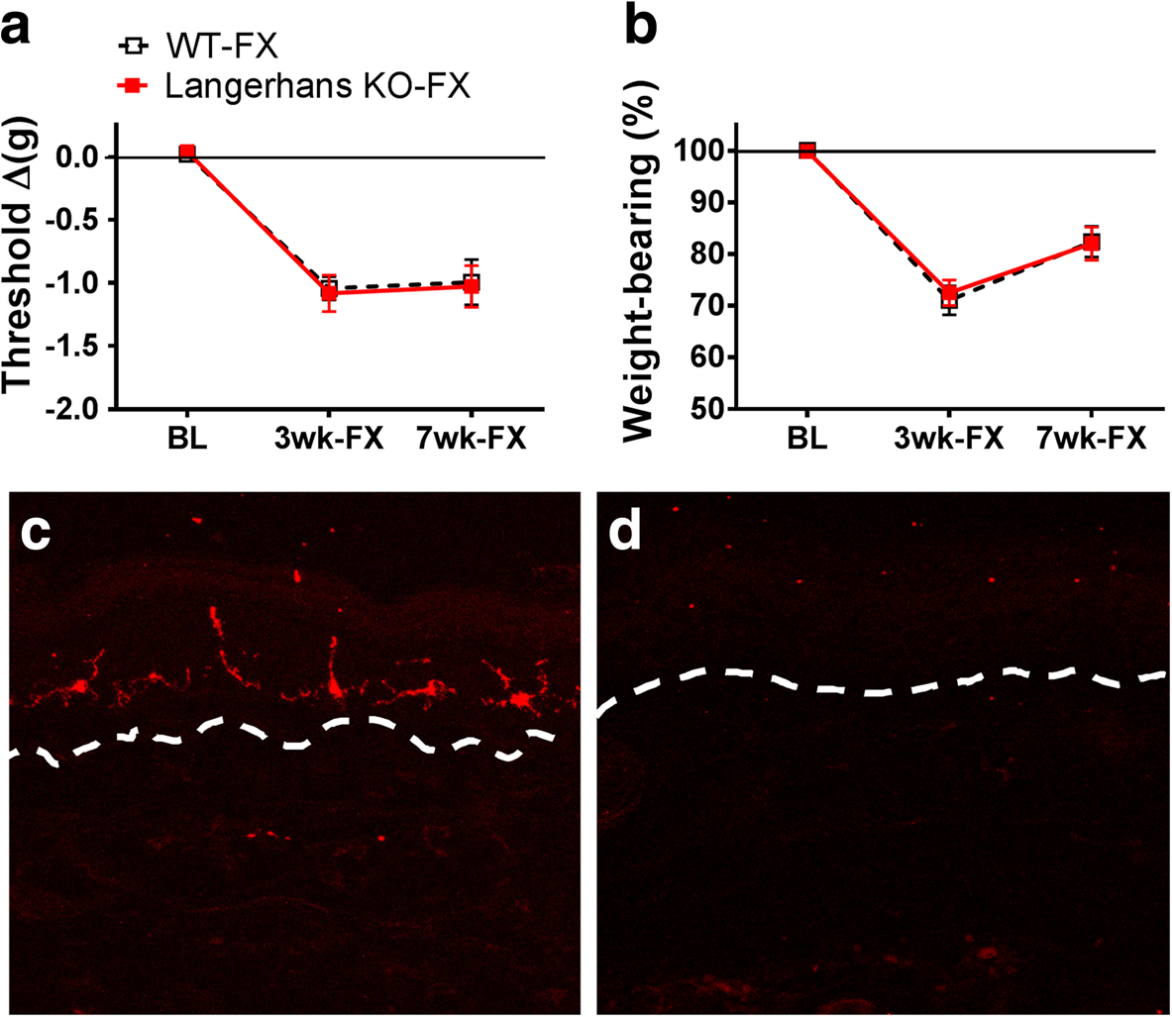
Epidermal Langerhans cell (LC)-deficient fracture mice (Langerhans KO-FX) developed von Frey mechanical allodynia (**a**) and hindlimb unweighting (**b**) that did not differ from nociceptive changes observed in wildtype fracture mice (WT-FX) at 3 and 7 weeks after fracture. At the end of the behavioral testing, hind paw skin from WT-FX and Langerhans cell KO-FX mice were evaluated by immunohistochemistry. Langerin/CD-207 (red) was detected in frozen sections from WT-FX mice (**c**), but not in sections from Langerhans cell KO-FX mice (**d**). Dotted line indicates the epidermal-dermal boundary. Data were analyzed using a two-way analysis of variance (ANOVA), data are expressed as mean values ± SEM, *n* = 8–12 per cohort

The above findings led us to investigate the functional role of LCs in the development of nociceptive changes observed in the CRPS fracture model. To address this, LC-deficient mice were used. As showed in Fig. 10, compared to wildtype fracture mice, LC-deficient fracture mice exhibited similar CRPS-like nociceptive changes at 3 and 7 weeks after fracture. At the end of the behavioral testing, hind paw skin from wildtype and Langerhans cell-deficient fracture mice were harvested to identify LCs by immunohistochemistry. As expected, there were no Langerin-positive staining cells observed from the skin of the LC-deficient fracture mice. We went on to deplete LCs (and additional phagocytic cells, including other types of dendritic cells and macrophages [41]) using repeated intravenous liposomal clodronate injections. We observed that wildtype fracture mice treated with weekly intravenous or intradermal injections of encapsulated clodronate had mechanical withdrawal and unweighting results indistinguishable from wildtype fracture mice that received control liposomes, and these treatments did not reduce IgM antibody-antigen complex deposition in the fracture hind paw skin (data not shown). These results suggest that LCs (or other phagocytic immune cells) are not required for the development of nociceptive changes after fracture despite their activation by neuropeptides.

### Neuropeptide signaling regulates autoantigen expression after fracture

**Fig. 11:**
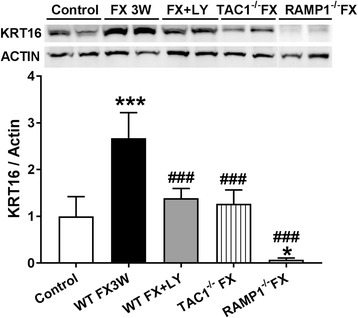
Neuropeptide signaling regulates the post fracture increase in cytokeratin 16 (Krt16) autoantigen expression in fracture limb skin. Western blot analysis demonstrated increased hind paw skin Krt16 levels at 3 weeks after fracture in wildtype fracture mice (WT-FX), vs no fracture controls (WT-NO FX). This increase was blocked by LY303870 treatment and in mice deficient in SP expression (Tac1^−/−^) after fracture. And Krt16 was found low levels in CGRP receptor signaling-deficient fracture mice (RAMP1^−/−^-FX). Krt16/actin band intensity ratio was calculated to compare expression levels between the groups. Data were analyzed using a one-way analysis of variance (ANOVA) with Bonferroni correction test for post hoc contrasts, data are expressed as mean values ± SEM, *n* = 4 per cohort. *** *P* < 0.001 vs. WT-NO FX, ### *P* < 0.001 vs. WT-FX

Failing to find evidence that neuropeptides regulate post-fracture autoimmunity via regulation of antigen processing by LCs or phagocytic immune cells (Fig. 10), we hypothesized that expression of autoantigens might be diminished in fracture mice lacking SP or CGRP signaling, therefore explaining the reduction in IgM antibody-antigen complex deposition in the ipsilateral hind paw skin, nerve, and cord of NK1 antagonist LY303870-treated WT and RAMP1^−/−^ fracture mice (Fig. 2), and the lack of pronociceptive effects observe with the serum autoantibodies obtained from mice with impaired neuropeptide signaling (Fig. 3). Previous work using 2-d gels of hind paw skin homogenates probed with fracture and control mouse sera and further analyzed by liquid chromatography-mass spectroscopy demonstrated unique binding between fracture sera and fracture paw skin Krt16 protein, elevated expression of Krt16 in fracture paw skin, and increased mouse fracture sera and CRPS patient sera binding to recombinant Krt16 protein [25]. Consistent with the hypothesis that Krt16 autoantigen expression is dependent on facilitated neuropeptide signaling after fracture, Figure 11 shows the elevation of Krt16 expression in hind paw skin 3 weeks after fracture in WT mice, but not in tissues from RAMP1^−/−^, Tac1−/− animals, or mice treated with the NK1 receptor antagonist LY303870.

## Discussion

The sometimes erythematous, warm, and swollen appearance of CRPS limbs has led to the discovery of neurogenic inflammation as supporting CRPS, with evidence of exaggerated SP and CGRP signaling in the affected skin [4, 13]. At the same time, a significant body of clinical research has demonstrated that the innate system of immunity is activated in CRPS patients resulting in the accumulation of mast cells, cytokines, and additional mediators in the limbs of CRPS patients [42, 43]. Laboratory studies using the tibia fracture-cast immobilization rodent model of CRPS have shown that SP and CGRP can stimulate keratinocytes to produce these inflammatory mediators in the fracture hindlimb skin [13, 44] during the first few months after injury [45], similar to the time course observed in CRPS-affected skin [42]. Recently, however, activation of the adaptive system of immunity has been implicated in CRPS [6, 46]. Interestingly, sensory nerve fibers are recognized to have key roles in regulating the adaptive in addition to the innate immune response [47, 48]. These observations make plausible the hypothesis that neuropeptides may support an autoimmune response after limb injury underlying the development and maintenance of CRPS.

In this set of investigations, we developed evidence showing (1) that intact SP and CGRP signaling are required for the production of autoantibodies supporting the CRPS-like changes characteristic of the mouse fracture-cast model, (2) that SP and CGRP signaling are required for the increases in LC numbers seen after fracture as well as production of several cytokines involved in LC recruitment and activation, (3) that despite increased numbers of LCs and their stimulatory cytokines, these cells are not required for the nociceptive sensitization seen after fracture, and (4) that SP and CGRP are required for the enhanced production of at least one confirmed target of CRPS-related autoantibodies, Krt16.

At this point, a diverse set of both clinical and laboratory observations characterize the likely roles autoimmunity plays in CRPS. For example, genetic studies link CRPS to sequence variants at specific HLA gene loci, genes involved in adaptive immunity [49, 50]. Antinuclear antibodies are found in a disproportionately large fraction of patients with CRPS [51]. Additional data exist showing that a subset of patients treated with weekly plasmapheresis to lower autoantibody levels experienced prolonged symptom improvement, although a recent large randomized trial with low-dose intravenous immunoglobulin (IVIG) was ineffective [52–54]. Studies using sera and purified IgG preparations from CRPS patients have identified sympathetic neurons, muscarinic receptors, and alpha-1 adrenergic receptors as targets of these autoantibodies [6, 20]. More recently, laboratory studies have shown that B cell knockdown or genetic deletion in muMT mice reduces the nociceptive sensitization seen in mice after tibia fracture and cast immobilization [23]. Furthermore, the passive transfer of sera or purified IgM from fracture mice to B cell-deficient muMT fracture mice can restore the full CRPS-like syndrome in the muMT animals [24]. This susceptibility to the effects of passively transferred autoantibodies lasts for more than 5 months after fracture in the muMT fracture mice. Likewise, the transfer of purified IgG from human CRPS patients to rats with hind paw incisions exacerbates the nociceptive sensitization seen in those animals [55]. To this point, however, there has been very little information helping us to understand how the adaptive system of immunity might become activated and maintain that level of activation. Here we used two very well characterized neuropeptide signaling-deficient transgenic animal models in addition to a highly selective pharmacological tool, LY303870, to block SP signaling. Data using these models uniformly suggests both SP and CGRP are required for the formation of nociception-supporting IgM autoantibodies after fracture in the mouse fracture-cast model.

During these studies, a substantial effort was made to pursue professional antigen presenting cells, namely LCs, as critical components of the innate immune activation in this model. These cells are of interest as the processing and presentation of antigens to B cells is a crucial step in the generation of antibodies [56]. A histological study of five CRPS patient skin biopsies using nonspecific immunostaining for LCs and historical controls reported elevated LC numbers in affected skin [21]. More recently, however, examination of Langerhans cell numbers in 10 patients with very chronic CRPS (average duration 5.5 years) showed a decrease in LC numbers [22]. When evaluated at 3 weeks after injury, as in the present studies, the fracture-cast immobilization model most closely resembles the acute or “warm” phase of CRPS with hind paw nociceptive sensitization, edema, and warmth and with high levels of skin inflammatory mediators and increased LC numbers (Figs. 4, 5, and 6). The human biopsies we examined were also from patients with relatively acute (< 24-week duration) CRPS, and LC numbers were increased in the skin biopsy from the affected limb, compared to the contralateral limb control skin biopsy (Fig. 7). We did not specifically assess the association of Langerhans cell numbers with skin temperature elevation. Furthermore, the enhanced levels of LCs and stimulatory cytokines were reduced in animals deficient in SP or CGRP signaling. Surprisingly, nociceptive sensitization was indistinguishable between the wildtype and LC knockout animals after fracture (Fig. 10). Similarly, depletion of LCs (and other phagocytic cells) with liposomally encapsulated clodronate also failed to alter nociceptive sensitization after fracture. We cannot conclude, however, that the regulation of antigen presentation is not important in supporting the autoimmune response seen in this model. B cells, for example, are considered professional antigen-presenting cells and may be responsible for autoreactive T cell activation and subsequent B cell autoantibody production as in the cases of lupus and multiple sclerosis [57]. Contrariwise, synovial peripheral helper T cells in rheumatoid arthritis patients are also effective antigen-presenting cells capable of inducing B cell autoantibody responses [58]. Moreover, our understanding of how “atypical” antigen presenting cells can initiate autoimmune responses is a very active area of research [59]. Such immunological processes may not be blocked by encapsulated clodronate.

The serum transfer experiments and immunoblotting results are consistent with the hypothesis that neuropeptide-dependent autoantibody production contributes to nociceptive sensitization in the fracture-cast model. These findings reproduce and extend previous reports focused on the roles of B cell activation and autoantibody production in fracture-cast mice [23, 25], and autoantibody production in human CRPS patients [6, 20]. They replicate earlier results demonstrating persistent IgM accumulation in the skin and nerves of the fracture limb and ipsilateral lumbar spinal cord after tibia fracture and casting [24]. Our findings also suggested that IgM autoantibodies under the control of neuropeptides are directed at Krt16, a keratin protein expressed in high abundance in epithelia, particularly the skin. This was selected as our index autoimmune target as anti-Krt16 antibodies have been identified both in our mouse model and in human CRPS patients [25]. However, we do not know if this target is functional in supporting nociceptive sensitization. Other human diseases characterized by autoimmunity and pain such as rheumatoid arthritis have many identified autoantigens, and in fact, our own experiments suggested that other keratin species, tubulin, alpha-enolase, peripherin, and other proteins may serve as autoantigens [25]. Stronger evidence for the production of functionally important CRPS-related autoantibodies comes from experiments where exposure of cultured myocytes to IgG purified from CRPS patients caused alterations in cultured myocyte contraction, and from companion experiments in which interactions of CRPS IgG with cells expressing M-2 muscarinic or β-2 adrenergic receptors caused intracellular calcium transients [20]. Many other such interactions supporting pain in CRPS are possible. For example, autoantibodies directed at the myelin sheaths of peripheral nerves support pain in Guillain-Barré syndrome, and a rare syndrome of autoimmunity against a potassium ion channel complex may be responsible for the pain and other sensory symptoms reported by these patients [46, 60]. It should also be recognized that autoantibodies need not directly modulate the activity of the target proteins to cause pain and inflammation. Immunoglobulins, particularly IgM bound to target tissues can activate the complement cascade through the classical pathway, and in fact, it was shown previously that autoantibody-mediated complement system activation was present in CRPS model mice [23]. In addition, rheumatoid arthritis patients express IgG autoantibodies to citrullinated proteins that have pronociceptive effects when injected into normal mice, mediated by osteoclast activation and release of the nociceptive IL-8 chemokine [61].

While the results of this study add to our knowledge about the activation of the immune system after trauma, we still lack a great deal of information. For example, we have examined neuropeptide actions, but did not explore the role of the sympathetic nervous system. The sympathetic nervous system has long been known to support CRPS, and sympathetic fibers innervate organs with important immune functions such as the spleen [62]. On the clinical level, we do have available potent, selective well-tolerated NK1 receptor antagonists such as aprepitant that might be used in clinical trials attempting to prevent CRPS after high-risk surgeries or perhaps speed the resolution of acute CRPS. Although the side effect profiles of immunosuppressant drugs are significant, medications that reduce B cell numbers interfere with T cell-B cell interactions or otherwise reduce adaptive immune responses may also prove useful. This work also suggests that screening for autoreactive IgM might provide a useful biomarker for cases of CRPS with a strong immune contribution. Regardless of the approach taken, the results of the current study support the premise of the immune system as a new disease-modifying target in CRPS.

## Conclusions

Limb fracture followed by cast immobilization leads to long-lasting nociceptive sensitization. This period of sensitization is accompanied by the accumulation of IgM in the skin and neural tissues serving the injured limb and the presence of pronociceptive serum antibodies. Pronociceptive IgM production is reliant upon intact neuropeptide signaling. It appears that neuropeptide signaling may be more strongly related to the enhanced expression of CRPS-related autoantigens than the stimulation of antigen processing. These findings open the door to the design of anti-SP and anti-CGRP signaling strategies for reducing post-traumatic autoimmunity.

## Abbreviations

ANOVA: Analysis of variance
CGRP: Calcitonin gene-related peptide
CRPS: Complex regional pain syndrome
FX: Fracture
IVIG: Intravenous immunoglobulin
Krt16: Keratin type 16
LC: Langerhans cell
LY: LY303870, a substance P tachykinin 1 receptor antagonist
NGF: Nerve growth factor
PBS: Phosphate buffered saline
PFA: Paraformaldehyde
SEM: Standard error of the mean
SP: Substance P
TBST: Tris-buffered saline with Tween
WT: Wildtype
